# Supplementary material for: SDS Depletion from Intact Membrane Proteins by KCl Precipitation Ahead of Mass Spectrometry Analysis
Source: Proteomes. 2025 Jul 2;13(3):30. doi: 10.3390/proteomes13030030 (PMC12286100; doi:10.3390/proteomes13030030)
Supplement: Supplementary file 1 [file proteomes-13-00030-s001.zip › Supporting Figures_revised.pdf]

proteomes

MDPI

SUPPORTING DATA FOR:

SDS Depletion from Intact Membrane Proteins by KCl Precipitation Ahead of Mass Spectrometry Analysis

Tania Iranpour, Mapenzi Mirimba, Chloe Shenouda, Adam Lynch and Alan A. Doucette *

Department of Chemistry, Dalhousie University, 6243 Alumni Crescent, Halifax, NS B3H 4R2, Canada; tn315509@dal.ca (T.I.); mp633299@dal.ca (M.M.); ch976052@dal.ca (C.S.); ad647601@dal.ca (A.L.)
* Correspondence: alan.doucette@dal.ca

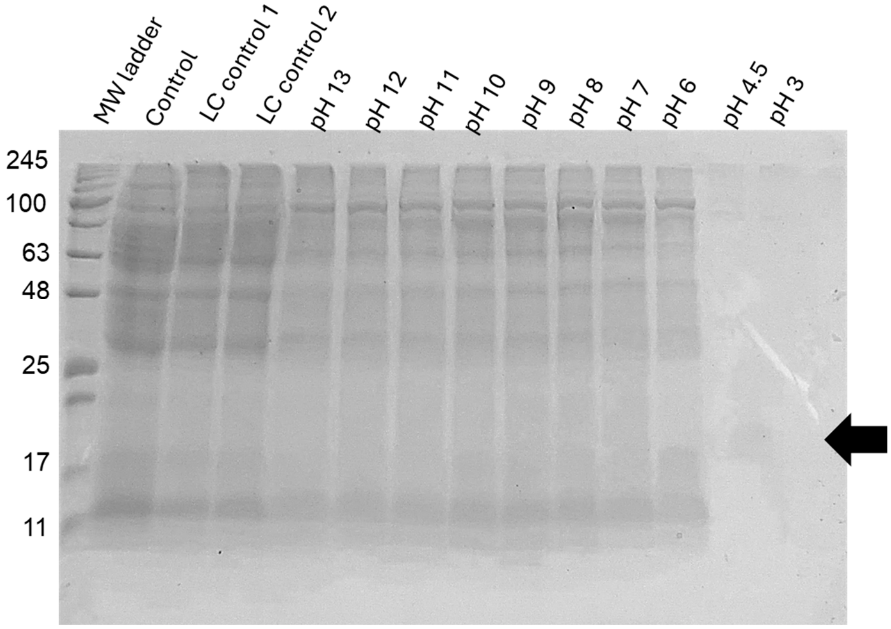

Figure S1. SDS PAGE image of liver total proteome extract subject SDS precipitation with KCl over a range of pH values. Control represents the original sample prior to addition of salt, urea or pH adjustment, while LC controls (1&2) are of the control sample following LC cleanup and reconstitution in gel buffer. The arrow indicates a faint band of low molecular weight proteins, still evident in the sample precipitated at pH 4.5.

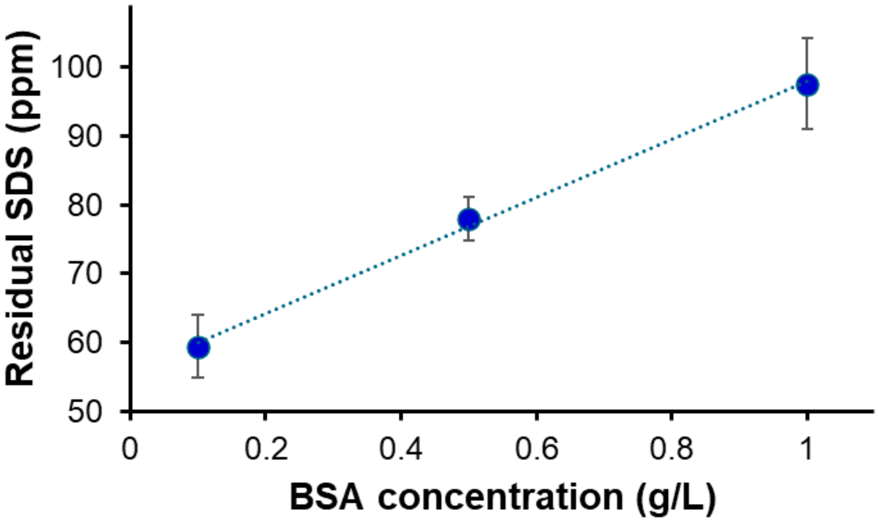

Figure S2. SDS precipitation by KCl as a function of the initial protein concentration. Increasing the amount of protein in the initial sample causes a higher concentration of residual SDS. However, a 10-fold increase in concentration does not cause a corresponding 10-fold increase in SDS. From this, it would be preferred to precipitate SDS from a highly concentrated protein sample, and dilute the resulting sample following precipitation. This would result in a lower ratio of protein to SDS (ie a more pure sample).

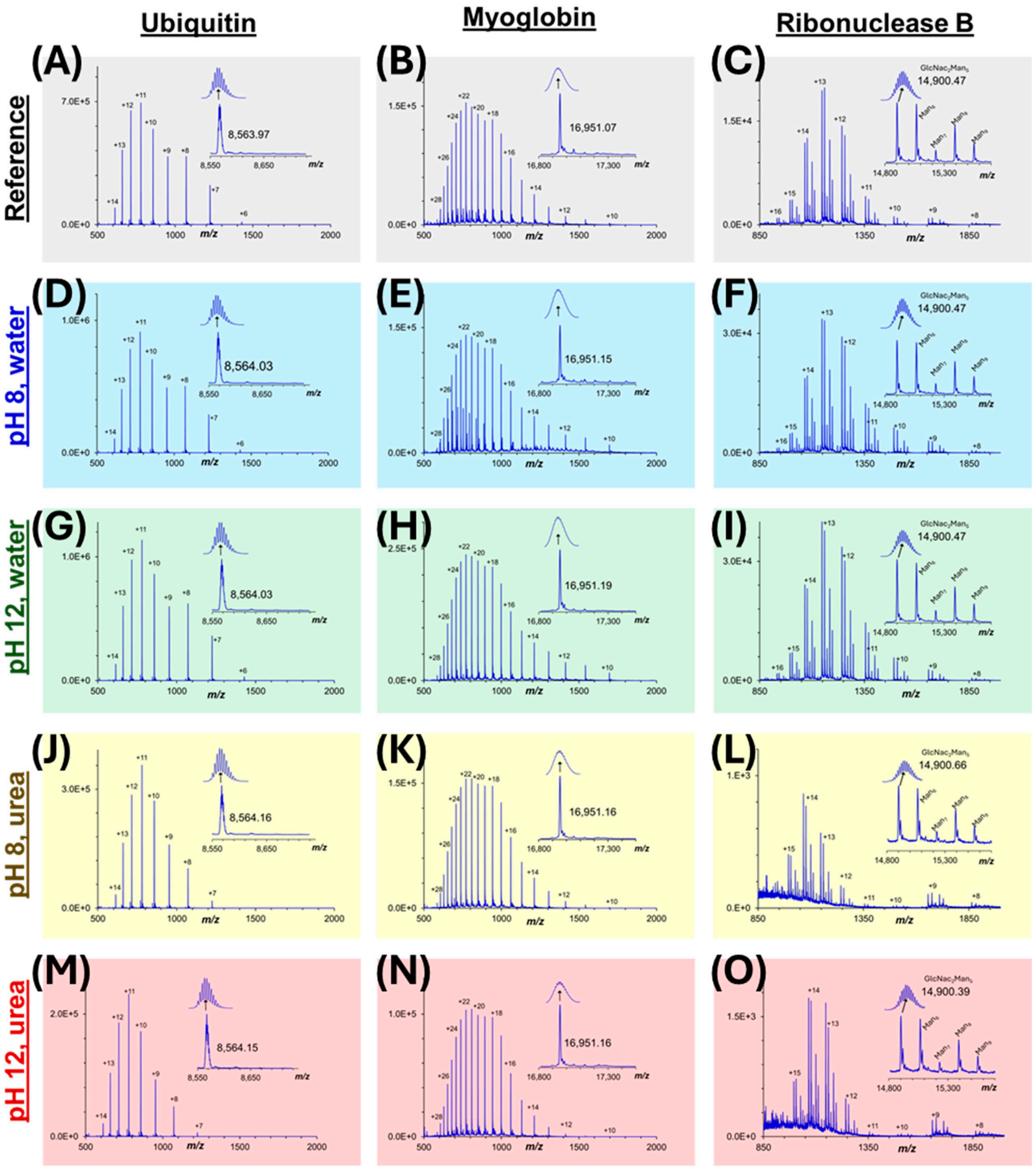

Figure S3. Charge envelope MS spectra of three intact protein standards (ubiquitin, myoglobin, ribonuclease B) are shown together with the deconvoluted MS spectra (inset). An expanded view of the deconvoluted protein peak is also provided (resolution ~10,000 FWHM). Panels **(A)**, **(B)** and **(C),** showing the MS spectra in grey, represent control samples, prepared at the equivalent concentration but in the absence of SDS. All other samples were recorded following the KCl depletion of SDS (initially 0.5%), in water at pH 8 (blue spectra), at pH 12 (green), in 8 M urea at pH 8 (yellow), and 8 M urea, pH 12 (red).
